# Editorial: Immune adaptations in aquatic species: defenses, gene diversity, and environmental stressors

**DOI:** 10.3389/fimmu.2026.1878159

**Published:** 2026-05-19

**Authors:** Antonio Figueras, Magalí Rey-Campos, Cristian Gallardo-Escárate, Valentina Valenzuela-Muñoz, Jorge Galindo-Villegas

**Affiliations:** 1 Instituto de Investigaciones Marinas-CSIC, Vigo, Spain; 2 Universidad de Concepción, Concepción, Chile; 3 Genomics, Nord University, Bodø, Norway

**Keywords:** aquatic organisms, genomics, global change, immunology, stress

Aquatic organisms inhabit some of the most immunologically demanding environments on Earth. Pathogens are pervasive, temperature fluctuates, pollution accumulates, and anthropogenic pressures are intensifying rapidly. Yet most aquatic invertebrates, which constitute the overwhelming majority of marine and freshwater biomass, mount their defenses without adaptive immunity as classically defined in vertebrates. Understanding how innate and constitutive immune mechanisms cope with these pressures, how genetic diversity underpins immune flexibility, and how environmental stressors compromise or reshape these systems has become a central question in comparative and environmental immunology.

This Research Topic assembles ten original investigations that address immune function across a broad taxonomic range, from decapod crustaceans and bivalve molluscs to cetaceans, pinnipeds, and marine mammals, and at multiple levels of biological organization: molecular, cellular, transcriptomic, and ecological.

## Environmental stressors and immune disruption

Three contributions address how physical stressors impair immune function. Duan et al. characterize the cascade of immune dysfunction and metabolic remodeling in the hepatopancreas of *Litopenaeus vannamei* under high-temperature stress, documenting a coordinated transcriptomic transition in one of global aquaculture’s most economically critical species. Chapuis et al. document a previously unreported consequence of anthropogenic underwater noise: suppression of the immune response in *Mytilus* spp. following *Vibrio splendidus* challenge, with direct implications for disease susceptibility in coastal ecosystems exposed to shipping and offshore construction. Zhang et al. offer a contrasting, adaptive perspective: in the red swamp crayfish *Procambarus clarkii*, non-lethal heat shock activates SOD gene family diversity and enhances resistance to subsequent bacterial challenge, identifying a hormetic immune-priming mechanism with potential relevance to thermal acclimation strategies in crustacean aquaculture.

## Host–pathogen interactions in bivalves

The bivalve–*Vibrio* axis receives focused attention from two complementary studies. Leonessi et al. examine interactions between the oyster larvae pathogen *Vibrio ostreicida* and two commercially important hosts, *Mytilus galloprovincialis* and *Magallana gigas*, characterizing differential susceptibility and immune activation at the larval stage. A companion study by Leonessi et al. tracks the dynamics of *V. ostreicida* infection in *M. galloprovincialis* through *in vivo* experimental challenges, providing time-resolved data on pathogen clearance and immune persistence that were previously unavailable for this emerging pathosystem.

## Cellular immunity and the hemocyte as an unconventional actor

Two papers address hemocyte biology from distinct vantage points. Abdallah et al. provide the first transcriptomic characterization of mucosal hemocytes in the eastern oyster *Crassostrea virginica*, demonstrating that this anatomically distinct population exhibits an immune-sentinel phenotype adapted to the mucosal interface, suggesting a functional division of labor among hemocyte types. Rey-Campos et al. describe a system of externalized immune surveillance in marine bivalves: immune sentinel hemocytes (ISCs) that migrate from the hemolymph into the intervalvar cavity and surrounding seawater, where they remain viable, exhibit immune-activated transcriptomic profiles, resist apoptosis, and lack allorecognition — properties that enable transfer between individuals. Their transcriptomic profiles partially overlap with transmissible neoplastic hemocytes while retaining a distinct functional immune identity. This describes a form of population-level immunity with no established precedent in the invertebrate literature.

## Transcriptomics of understudied vertebrates

Two studies apply transcriptomics to non-model vertebrate species where immune data remain scarce. Dönmez et al. characterize immune gene expression in the lung and muscle of harbor porpoises (*Phocoena phocoena*) presenting pathological conditions, providing a baseline for interpreting immune responses in a cetacean species facing increasing anthropogenic pressure in coastal waters. Qin et al. address a fundamental gap in pinniped immunogenomics by annotating and comparatively analyzing T cell receptor germline genes across pinniped lineages, revealing lineage-specific diversification patterns that reflect both phylogenetic history and ecological context.

## Epigenetic regulation and local adaptation

The final contribution extends the scope beyond classical immunity. Yévenes et al. use reciprocal transplantation experiments in *Mytilus chilensis* to identify differential expression of long non-coding RNAs associated with local adaptation, demonstrating that epigenetic mechanisms, not only coding sequence variation, contribute to the phenotypic flexibility that enables survival across heterogeneous marine environments.

## Synthesis

Taken together, these ten studies show that immune function in aquatic species is not static, not confined to the organism’s interior, and not comprehensible through the lens of vertebrate models alone. The externalized ISC system in bivalves, the hormetic SOD response in crustaceans, the noise-mediated immune suppression in mussels, and the epigenetic flexibility documented in *M. chilensis* each challenge assumptions that have persisted in comparative immunology for decades. The vertebrate contributions from pinnipeds and cetaceans underscore how much basic immunogenomic information remains absent, even for well-studied marine mammals.

Climate change and coastal degradation will continue to reshape the pathogen landscape and thermal regimes that aquatic species face. The molecular and cellular mechanisms described in this Research Topic constitute the biological substrate on which those pressures will act. Understanding them on their own terms, rather than as approximations of mammalian immunity, is the prerequisite for any credible prediction of how aquatic ecosystems will respond.

The Guest Editors thank all authors for their contributions and all reviewers for the rigor of their assessments.

